# Сoping with hostility in suicidal ideation in mental illness and control groups

**DOI:** 10.1192/j.eurpsy.2026.12080

**Published:** 2026-07-31

**Authors:** T. I. Medvedeva, O. Y. Vorontsova, O. M. Boyko, S. N. Enikolopov, S. O. Kuznetsova, M. I. Oleychik, D. S. Bichevina, P. I. Varvara

**Affiliations:** 1 clinical psychology, Federal State Budgetary Scientific Institution Mental health scientific center; 2 State Budgetary Healthcare Institution “Moscow City Center for the Reabilitation of Patients with Spinal Cord Injury and Consequences of Infantile Cerebral Palsy Department of Healthcare”, Moscow, Russian Federation

## Abstract

**Introduction:**

Suicidal thoughts are often associated with high levels of hostility and self-harm. Using effective ways of coping with aggression and hostility can reduce the risk of suicide.

**Objectives:**

The aim was to analyse the relationship between the suicidal ideation and legitimate hostility coping in mental illness and healthy groups.

**Methods:**

Рatients from a mental health clinic (N=75, 12% male, mean age 21.9±4.8) with depressive spectrum disorders and a control group (N=112, 25% male, mean age 19.8±3.7).

Each group was divided into two subgroups – ‘with suicidal ideation’ and ‘without suicidal ideation’ – based on the severity of suicidal ideation (Likert scale).

«Emotional coping» was assessed using CTI (Epstein). The acceptability of harming others for the ‘common good’ was assessed using Moral Dilemmas (Greene). The acceptability of aggression in personal relationships was assessed using the Legitimised Aggression Questionnaire based on PLAQ (Hogben). BPAQ-24, SCL-90-R were used. Autoaggressive tendencies were assessed using questions about alcohol use and self-harm.

**Results:**

Correlation analysis showed that in both the clinical and control groups, the severity of suicidal intentions was associated with higher manifestations of hostility (SCL-90R, BPAQ-24), manifestations of autoaggressive behaviour (alcohol, self-harm), and reduced emotional coping ability (CTI).

Subgroup analysis showed that in the presence of suicidal ideation, the degree of hostility and autoaggressive manifestations did not differ between the clinical and control subgroups. However, emotional coping in the clinical group was significantly lower (Fig. 1).

For the control group, suicidality is characterised by a more frequent choice to risk the lives of others (in moral dilemmas), which may indicate a choice of legitimised forms of aggression. No such connection was found for the clinical group. Suicidal thoughts in the clinical group are associated with lower legitimised aggression in personal life, upbringing and politics; no such connection was found in the control group (Fig. 2).

**Image 1:**
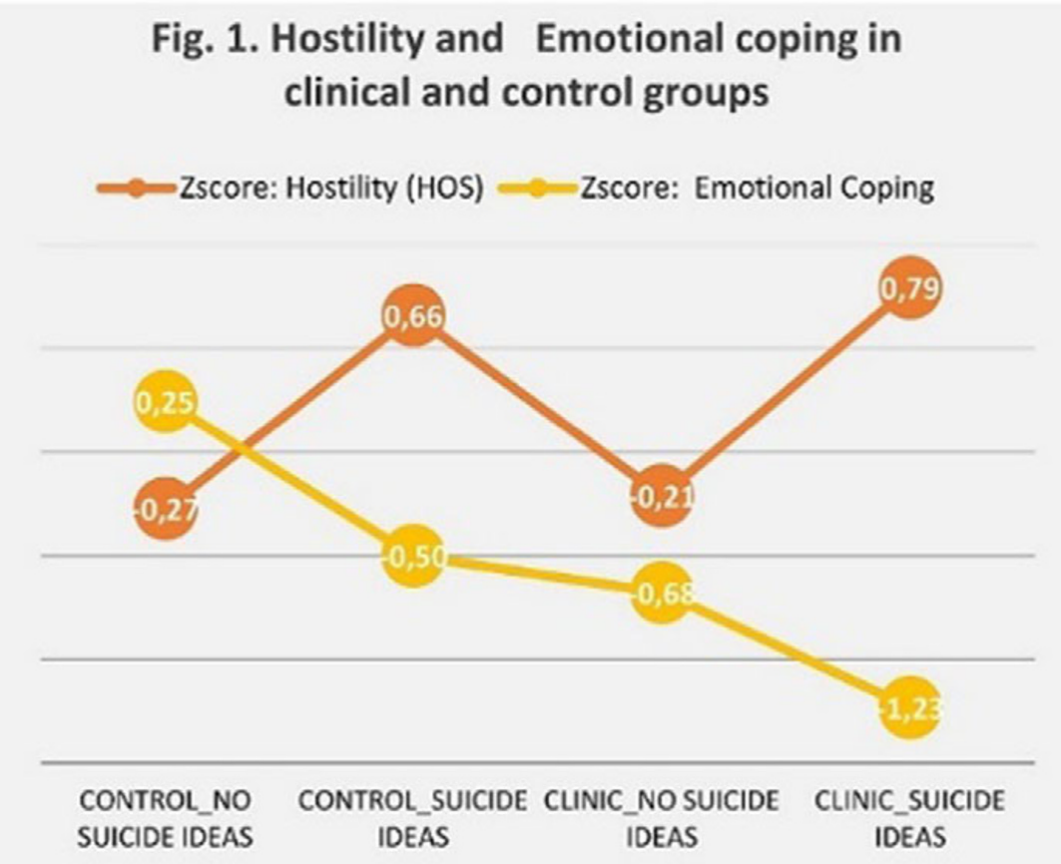
Image 1: Long description.

**Image 2:**
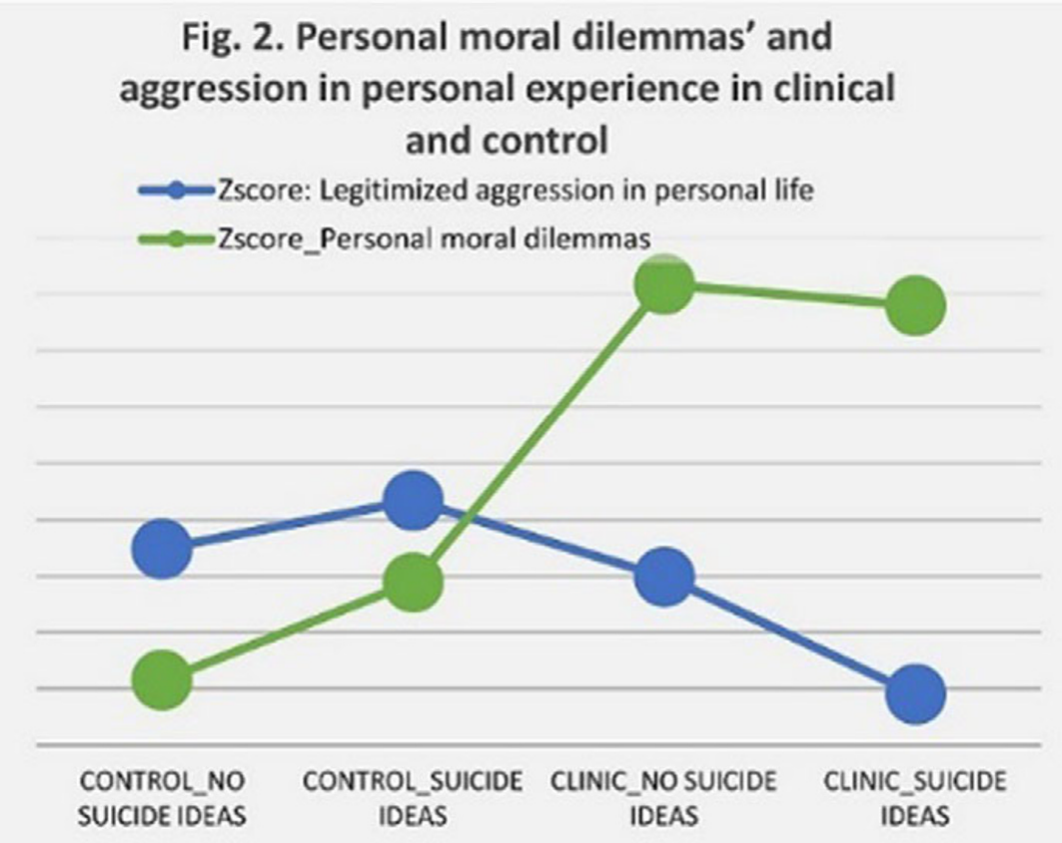
Image 2: Long description.

**Conclusions:**

Common factors related to suicidal ideation were identified for the clinical group and the group of healthy subjects, including hostility and autoaggressive tendencies. The difference between the groups is related to the ability to express one’s hostility towards others in a legitimate way. In the control group, suicidal tendencies are associated with more frequent decisions that put the lives of others at risk. In contrast, in the clinical group, an increase in the intensity of suicidal thoughts is not associated with a readiness to use legitimized ways of expressing aggression towards others, and even shows a decrease in the use of legitimized aggression in personal experience, upbringing, and politics.

**Disclosure of Interest:**

None Declared

## Long descriptions

### Image 1: Long description

The X axis lists four categories from left to right. CONTROL_NO SUICIDE IDEAS, CONTROL_SUICIDE IDEAS, CLINIC_NO SUICIDE IDEAS, and CLINIC_SUICIDE IDEAS. The Y axis represents Z scores with horizontal grid lines.

Two data series are plotted.

1. Zscore. Hostility H O S, represented by an orange line with circular markers.

- CONTROL_NO SUICIDE IDEAS. -0,27

- CONTROL_SUICIDE IDEAS. 0,66

- CLINIC_NO SUICIDE IDEAS. -0,21

- CLINIC_SUICIDE IDEAS. 0,79

The trend shows a sharp increase between the control groups, a drop for the clinic group without suicide ideas, and a peak for the clinic group with suicide ideas.

2. Zscore. Emotional Coping, represented by a yellow line with circular markers.

- CONTROL_NO SUICIDE IDEAS. 0,25

- CONTROL_SUICIDE IDEAS. -0,50

- CLINIC_NO SUICIDE IDEAS. -0,68

- CLINIC_SUICIDE IDEAS. -1,23

The trend shows a continuous linear decrease across all four groups from left to right.

Navigate back to Image 1:.

### Image 2: Long description

The x-axis contains four categories from left to right: CONTROL_NO SUICIDE IDEAS, CONTROL_SUICIDE IDEAS, CLINIC_NO SUICIDE IDEAS, and CLINIC_SUICIDE IDEAS. The y-axis represents Z-score values indicated by horizontal grid lines.

There are two data series:

* Zscore: Legitimized aggression in personal life (blue line with circular markers): This series starts at a moderate level for the first control group, rises slightly for the second control group, then shows a steady linear decrease through the two clinical groups, reaching its lowest point at CLINIC_SUICIDE IDEAS.

* Zscore_Personal moral dilemmas (green line with circular markers): This series starts at a low level for the first control group, rises slightly for the second control group, and then exhibits a sharp, steep increase to a peak at CLINIC_NO SUICIDE IDEAS. It remains at a high level with a very slight decrease for the final CLINIC_SUICIDE IDEAS group.

The two lines intersect between the CONTROL_SUICIDE IDEAS and CLINIC_NO SUICIDE IDEAS categories, where the green line surpasses the blue line.

Navigate back to Image 2:.

